# Co-expression of CD30 and SLFN11 serves as a dual biomarker for the treatment of cutaneous T-cell lymphoma

**DOI:** 10.1093/narcan/zcaf037

**Published:** 2025-10-07

**Authors:** Takatoshi Shimauchi, Junko Murai, Manami Iwasaki, Pawit Phadungsaksawasdi, Toshiyuki Ojima, Kazuyasu Fujii, Tetsuya Honda

**Affiliations:** Department of Dermatology, Hamamatsu University School of Medicine, Hamamatsu, 431-3192, Japan; Division of Cell Growth and Tumor Regulation, Proteo-Science Center, Toon, Ehime, 791-0295, Japan; Department of Biochemistry and Molecular Genetics, Ehime University Graduate School of Medicine, Toon, Ehime, 791-0295, Japan; Department of Dermatology, Hamamatsu University School of Medicine, Hamamatsu, 431-3192, Japan; Department of Dermatology, Hamamatsu University School of Medicine, Hamamatsu, 431-3192, Japan; Department of Community Health and Preventive Medicine, Hamamatsu University School of Medicine, Hamamatsu, 431-3192 Japan; Department of Dermatology, Kindai University Hospital, Osaka, 589-8511, Japan; Department of Dermatology, Hamamatsu University School of Medicine, Hamamatsu, 431-3192, Japan

## Abstract

Advanced-stage cutaneous T-cell lymphoma (CTCL) is treated with diverse modalities, including DNA-damaging agents, anti-CD30 antibody–drug conjugates, and histone deacetylase (HDAC) inhibitors. Schlafen 11 (SLFN11) has emerged as a key determinant of sensitivity to DNA-damaging agents, yet its role in CTCL remains unclear. Here, we examined SLFN11 expression in two major CTCL subtypes—mycosis fungoides (MF) and Sézary syndrome (SS). Immunohistochemistry revealed SLFN11 positivity in 52% of MF (13/25) and 80% of SS (4/5) cases, with multivariate analysis showing a significant correlation between SLFN11 and CD30 expression. In normal human peripheral blood mononuclear cells, CD3/CD28/IL-2 stimulation induced co-expression of SLFN11 and CD30 in T cells, which was accompanied by heightened sensitivity to DNA-damaging agents. The JAK inhibitor cerdulatinib suppressed both markers. Among five CTCL cell lines, HUT78—expressing the highest SLFN11 levels—was the most sensitive to DNA-damaging agents, whereas SLFN11 knockout conferred resistance. Attempts to restore SLFN11 expression in SLFN11-low CTCL cells using six (pre)clinical HDAC inhibitors produced inconsistent results across cell lines and drugs. Together, these findings identify SLFN11 and CD30 as co-expressed therapeutic targets in CTCL and support the rationale for CD30-directed antibody–DNA-damaging agent conjugates as a precision treatment strategy.

## Graphical Abstract

**Graphical Abstract:**
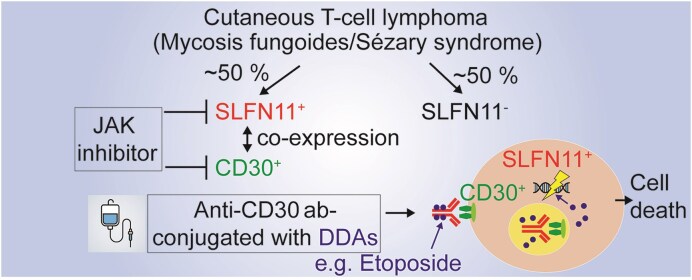

## Introduction

Cutaneous T-cell lymphomas (CTCLs) are a heterogeneous group of non-Hodgkin T-cell lymphomas that primarily affect the skin. Mycosis fungoides (MF) is the most common type of CTCL, accounting for >50% of cases [1, 2]. MF patients are classified into two groups: early stage (stage IA–IIA) and advanced stage (stage IIB–IVB) according to the TNMB classification [3]. Early-stage MF patients typically present with patches or plaques (T1–T2) with or without minimal peripheral blood (B0–B1) or lymph node (N0–N2) involvement. In contrast, advanced-stage MF (tumor-stage MF) patients develop dome-shaped skin tumors (T3), skin ulcers, or erythroderma (T4), with or without peripheral blood (B0–B2), lymph node (N0–N3), or visceral involvement (M1). Sézary syndrome (SS) is a less frequent erythrodermic variant of CTCL and is categorized as advanced stage (stage IV) with peripheral blood (B2) and lymph node involvement (N1–N3) [3]. Early-stage MF has a good prognosis with a median survival of >15 years and a 5-year survival rate of over 80% [4–6]. In contrast, advanced-stage MF or SS has a poor prognosis with a median survival of 63 months and 2- and 5-year survival rates of 77% and 52%, respectively [7]. Notably, stage IV, age over 60 years, large-cell transformation, and increased lactate dehydrogenase (LDH) are independent prognostic factors for worse survival in patients with advanced-stage MF [7]. In general, serum values of LDH and soluble interleukin-2 receptor (sIL-2R) are considered useful markers for MF/SS patients.

For patients with advanced-stage MF/SS, various systemic therapies have been utilized, including retinoid X receptor agonists (bexarotene), histone deacetylase (HDAC) inhibitors (vorinostat or romidepsin), an antifolate (pralatrexate), single-agent chemotherapies [etoposide (ETP) or gemcitabine], or multi-agent chemotherapies (CHOP or THP-COP) [8–10]. More recently, novel biologic therapies have emerged, including mogamulizumab (a monoclonal antibody against CCR4), brentuximab vedotin [an anti-CD30 monoclonal antibody conjugated to the microtubule toxin monomethyl auristatin E (MMAE)], or denileukin diftitox (a recombinant cytotoxic fusion protein composed of the diphtheria toxin fragments A and B fused to human IL-2) [8–10]. Despite the heterogeneous responses to these treatments, predictive biomarkers for therapeutic efficacy remain to be elucidated.

Schlafen 11 (SLFN11), a nuclease with single-strand DNA-binding and helicase domains, is an emerging predictive biomarker in cancer chemotherapy as it sensitizes cancer cells to a broad range of DNA-damaging anticancer agents, such as topoisomerase I (TOP1) inhibitors [e.g. camptothecin (CPT)], TOP2 inhibitors (e.g. ETP), antimetabolites (e.g. gemcitabine), platinum derivatives (e.g. cisplatin), and PARP inhibitors (e.g. olaparib) [11–16]. Mechanisms underlying SLFN11-mediated cell death include its RNase activity targeting type II transfer RNAs [17–20], induction of lethal replication blocks through single-stranded DNA binding [21–25], and induction of TP53-independent apoptosis [20, 26]. The utility of SLFN11 as a drug-sensitive biomarker has been validated in breast [27], ovarian [28–30], stomach [31], bladder [32], lung [33, 34], esophagus [35], head and neck [36], prostate cancers [37], and a subset of medulloblastoma [38]. Intriguingly, SLFN11 expression is predominantly regulated through epigenetic mechanisms, allowing epigenetic modifiers, such as HDAC inhibitors, to restore SLFN11 expression and re-sensitize cancer cells to chemotherapeutic agents [31, 39, 40].

Currently, the expression status of SLFN11 and its regulatory mechanisms in MF/SS tumor cells are completely unknown. In this study, we investigated the association between SLFN11 expression and clinical parameters in MF/SS samples and further explored the functional roles and regulatory mechanisms of SLFN11 in MF/SS patients.

## Materials and methods

### Patients

Twenty-five MF and five SS patients were enrolled. Additionally, two patients with lymphomatoid papulosis (LyP), five with primary cutaneous anaplastic large cell lymphoma (pcALCL), and five with adult T-cell leukemia lymphoma (ATL) were included for the analysis of SLFN11 expression. All patients were diagnosed and treated at the Department of Dermatology, Hamamatsu University School of Medicine, between January 2010 and December 2021. Diagnoses were made according to the criteria of the International Society for Cutaneous Lymphomas (ISCL), the United States Cutaneous Lymphoma Consortium, and the Cutaneous Lymphoma Task Force of the European Organization for Research and Treatment of Cancer (EORTC) [3]. TNMB classification and staging of MF/SS followed the ISCL/EORTC guidelines [3]. Clinical data collected from MF/SS patients at skin biopsy included age, sex, type of skin manifestation, serum LDH, and sIL-2R levels. Additional data included the date of final TNMB classification/staging, systemic therapies administered before and after the final staging, survival status, and censoring information. Overall survival (OS) was calculated from the date of final staging until death from any cause. Patients who were alive at the end of follow-up were censored at the last known date they were alive before March 2025.

All studies adhered to the principles of the Declaration of Helsinki. Experimental protocols received approval from the medical ethics committees of Hamamatsu University School of Medicine (No. 20-084). Written informed consent was obtained from the patients, and if not, an opt-out option for the protocol was also made available to the public online at Hamamatsu University School of Medicine.

### Immunohistochemistry and immunofluorescence staining

A skin biopsy was performed on lesional skin, and the deparaffinized sections were stained with mouse anti-SLFN11 antibody (1:500; Santa Cruz Biotechnology, #sc-515071x clone D-2; 2 mg/ml) as described in a previous report [41]. Detection was carried out using Histofine^®^ Simple Stain Max PO (MULTI) and Histofine^®^ DAB-3S (both from Nichirei Biosciences). Additional markers, including CD4, CD8, and CD30, were also evaluated based on routine staining results in our hospital using the same sample. For quantification of SLFN11 and CD30 expression, the percentage of SLFN11- or CD30-positive cells was calculated with StrataQuest software (TissueGnostics). SLFN11 or CD30 was considered positive when at least 10% or 5% of the infiltrating cells tested positive, respectively.

For immunofluorescence staining of skin tissue, deparaffinized skin specimens were treated with DAKO Target Retrieval Solution pH 9 (DAKO, S2368) for 30 min at 95°C, then blocked with 10% donkey serum and 1% bovine serum albumin (BSA) in phosphate buffered saline (PBS) for 30 min at room temperature (RT). Sections were incubated with rabbit anti-CD30 antibody (1:200; Cell Signaling Technology, #54 535, clone E4L4I) and mouse anti-SLFN11 antibody (D-2) at RT for 1 h. After washing, the slides were incubated with matched secondary antibodies, Alexa Fluor 594 donkey anti-mouse IgG and Alexa Fluor 488 donkey anti-rabbit IgG (1:200; Thermo Fisher Scientific), for 30 min at RT, then washed and mounted with Mounting Medium with DAPI (Thermo Fisher Scientific). Samples were examined using ZEISS Celldiscoverer 7 (Carl Zeiss Microscopy GmbH) and imaged with ZEN software (Carl Zeiss Microscopy GmbH). Image cytometry analysis was performed using StrataQuest software.

For immunofluorescence staining of peripheral blood mononuclear cells (PBMCs), cells were placed onto slide glasses (Superfrost Plus Microscope Slides, precleaned, Fisher Scientific) using Cytospin (Fisher Scientific). The cells were fixed with 4% paraformaldehyde (Nacalai Tesque) in PBS for 10 min, then permeabilized with 0.5% Triton X-100/PBS for 15 min. They were incubated overnight at 4°C with primary antibodies, including rabbit monoclonal anti-CD30 antibody (Abcam, ab252561 clone BLR055F), rabbit monoclonal anti-CD3 antibody (Abcam, ab135372 clone SP162), or mouse monoclonal anti-SLFN11 antibody (D-2), diluted 1:300 in 4% BSA/PBS with 0.1% Tween 20 (PBS-T). Following washes with PBS-T, the cells were incubated for 2–4 h with suitable secondary antibodies diluted 1:1000 in 4% BSA/PBS-T. After additional washing with PBS-T, the cells were mounted with Mounting Medium containing DAPI (Abcam). Images were captured using a Zeiss LSM 900 or Zeiss LSM 780 confocal microscope (Carl Zeiss Microscopy GmbH). Throughout the procedure, slides were kept protected from light.

### Cell lines and normal human PBMCs

Five CTCL cell lines—HUT78, MyLa, MJ, SeAx, and HH—were used for *in vitro* experiments. HH and MJ were purchased from the American Type Culture Collection (Manassas, VA, USA), while the HUT78 cell line was obtained from the Institute of Development, Aging, and Cancer at Tohoku University (Miyagi, Japan). MyLa and SeAx were kindly provided by Prof. Dummer (Department of Dermatology, Zurich University, Switzerland). According to a previous report, MyLa cells represent advanced-stage MF skin tumor cells, HH cells are indicative of aggressive leukemic MF tumor cells, and SeAx and Hut78 cells are leukemic cells of SS. MJ cells are a leukemic form of ATL caused by infection with human T-cell leukemia virus type 1 [42]. All the CTCL cell lines were cultured in RPMI-1640 (FUJIFILM, Cat. No. 189-02145) supplemented with 2 mM l-glutamine, 10% fetal calf serum (FCS) (Hyclone), 100 IU/ml penicillin, and 100 μg/ml streptomycin (Nacalai Tesque, Cat. No. 26252-94).

Frozen human PBMCs (iQ Biosciences, IQB-PBMC102; 50 million cells per vial) were thawed and cultured in six-well plates using RPMI-1640 medium supplemented with 2 mM l-glutamine, 5% FCS, 100 IU/ml penicillin, and 100 μg/ml streptomycin. The PBMCs were either left untreated or treated with 20 ng/ml (400 U/ml) human IFN-γ (PEPROTECH, #300-02-20UG) or 30 U/ml human IL-2 (Roche, Cat. No. 11011456001) along with Dynabeads Human T-Activator CD3/CD28 (VERITAS, DB11131D) until harvesting.

### Western blot analysis

Whole-cell lysates were prepared using RIPA buffer, incubated at 4°C for 3 min, and then briefly centrifuged. The clarified samples were mixed with Sample Buffer containing 2-Mercaptoethanol (2×) for sodium dodecyl sulfate–polyacrylamide gel electrophoresis (Nacalai Tesque), heated at 95°C for 10 min, and loaded onto Mini-PROTEAN TGX Gels (Bio-Rad Laboratories). Proteins were transferred to polyvinylidene difluoride (PVDF) membranes using a Trans-Blot Turbo RTA Transfer Kit (Bio-Rad Laboratories). Membranes were blocked with 4% BSA in PBS-T for 1 h at RT and subsequently immunoblotted overnight at 4°C with primary antibodies, including rabbit monoclonal anti-CD30, anti-CD3, mouse monoclonal anti-SLFN11, rabbit monoclonal anti-Pan-Actin (Cell Signaling Technology), or mouse monoclonal anti-GAPDH (Cell Signaling Technology), all at 1:3000 dilution in 4% BSA/PBS-T. After incubation, membranes were treated with horseradish peroxidase (HRP)-conjugated secondary antibodies at 1:5000 dilution in 4% BSA/PBS-T. Protein detection was carried out using Clarity Max Western ECL substrate (Bio-Rad Laboratories) and visualized with an ImageQuant mini (Cytiva). Signal quantification was performed using Fiji software (NIH).

### Viability assay

Cells (5000 per well) were seeded into 96-well white plates (SPL Life Sciences, Cat. No. SPL-30196) using 100 μl of medium per well. Following a 48- to 72-h exposure to specified drug concentrations in triplicate, cell viability was evaluated employing an ATPlite 1step Kit (Perkin Elmer, Cat. No. 6016731). Briefly, 25 μl of the ATP luminescent reagent was added to each well, and after a 5-min incubation, luminescence was measured using either a FlexStation3 (Molecular Devices) or a Varioskan LUX multimode microplate reader (Thermo Scientific). The ATP signal from untreated control cells was normalized to 100%. The viability percentage of treated cells was computed as (ATP in treated cells / ATP in untreated cells) × 100.

### Establishment of SLFN11 knockout cells

This study describes the generation of SLFN11 knockout (KO) HUT78 cell lines, designated as #A and #B. The targeted disruption of the *SLFN11* gene using CRISPR/Cas9 technology has been previously reported [43]. Briefly, guide RNAs #A (5′-gcgttccatggactcaagag-3′) and #B (5′-gttgagcatcccgtggagat-3′) were cloned into the pX330 plasmid (Addgene_101733). Corresponding gene-targeting constructs, containing homology arms and a puromycin resistance cassette, were engineered and co-transfected with the pX330 vectors into HUT78 cells via electroporation (NEPAgene). Following transfection, cells were cultured in drug-free medium for 48 h, then subjected to selection with puromycin until single-cell colonies emerged. These clones were expanded, and successful gene knockouts were confirmed by western blot analysis. This research received approval from the Genetic Modification Committees at Ehime University (No. M23-16) and Keio University (No. 68), adhering to institutional guidelines.

### Drugs

CPT (Cayman Chemical Company, 11694), ETP (FUJIFILM Wako Pure Chemical Corporation, 055-08431), tucidinostat (Selleckchem, S8567), belinostat (Selleckchem, S1085), CUDC-101 (Selleckchem, S1194), entinostat (Selleckchem, S1053), panobinostat (Selleckchem, S1030), vorinostat (Selleckchem, S1047), and cerdulatinib (MedChemExpress, HY-15999) were purchased.

### Statistical analysis

All statistical analyses were performed using GraphPad Prism version 7 (GraphPad Software) or EZR (Saitama Medical Center, Jichi Medical University, Saitama, Japan). EZR is a graphical user interface for R (The R Foundation for Statistical Computing, Vienna, Austria), specifically designed as a modified version of R Commander to facilitate the use of statistical functions commonly employed in biostatistics [44]. The Pearson correlation coefficient was calculated to evaluate the relationships between the log-transformed percentages of SLFN11^+^ and CD30^+^ cells, serum sIL-2R levels, and SLFN11 and CD30 (TNFRSF8) messenger RNA expressions in cell lines from the Genomics of Drug Sensitivity in Cancer (GDSC) database. OS was analyzed via the Kaplan–Meier method, with differences assessed using the log-rank test. The association between SLFN11 expression and clinicopathologic parameters was examined using Fisher’s exact test (univariate analysis). Multivariate analysis employed a logistic regression model. Group comparisons in Fig. 4B and D were conducted using one-way ANOVA followed by Dunnett’s multiple comparison test. Significance levels were denoted as follows: *P* > .05 (not significant, N.S.), *P* < .05 (*), and *P* < .001 (***).

## Results

### Clinical characteristics of patients with MF/SS

**Figure 1.**
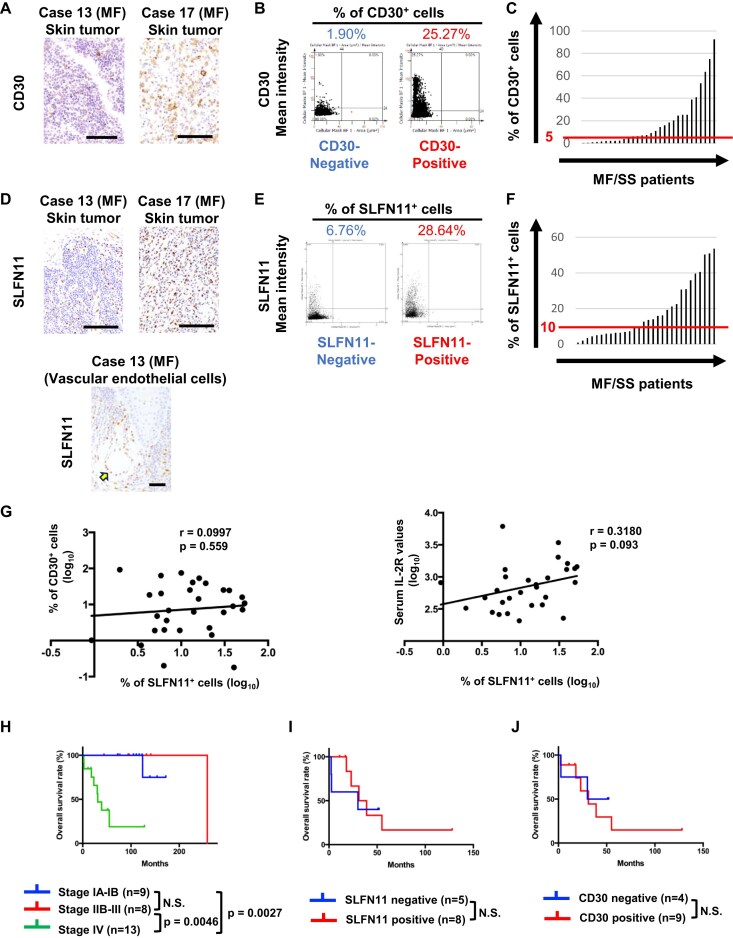
SLFN11 expression in MF/SS and its impact on clinical outcome. (**A**) Representative images of immunohistochemical staining for CD30 in skin-infiltrating MF tumor cells (original magnification, scale bar indicating 100 μm). (**B**) Quantification of CD30-positive population (%) in MF/SS tumors using an image analysis software (StrataQuest 7). Representative CD30-negative and -positive results are shown. (**C**) Distribution of CD30-positive population (%) in all cases of MF/SS. A red line indicates a cut-off value at 5%. (**D**) Representative images of immunohistochemical staining for SLFN11 in skin-infiltrating MF tumor cells (top). Vascular endothelial cells in the same sample from Case 13 are positive for SLFN11 (bottom, arrow; original magnification, scale bar indicating 100 μm). (**E**) Quantification of SLFN11-positive population (%) in MF/SS tumors using an image analysis software (StrataQuest 7). Representative SLFN11-negative and -positive results are shown. (**F**) Distribution of SLFN11-positive population (%) in all cases of MF/SS. A red line indicates a cut-off value at 10%. (**G**) Correlation analysis between % of SLFN11-positive (SLFN11^+^) cells and % of CD30-positive (CD30^+^) cells (right) or % of SLFN11^+^ cells and serum values of sIL-2R (left). Each dot represents one patient. The correlation coefficient (*r*) was calculated using the Pearson correlation coefficient test. (**H**) Kaplan–Meier curves for OS rate (%) in all the enrolled MF/SS patients (25 in MF and 5 in SS patients). For stage IA–IB versus IV, hazard ratio (HR), 0.1183; 95% confidence interval (CI), 0.02969–0.4714; *P*= .0027 (log-rank test). For stage IIB–III versus IV, HR, 0.1041; 95% CI, 0.02491–0.4350; *P*= .0046. For stage IA–IB versus IIB–III, HR, 5.755; 95% CI, 0.1096–302.1; *P*= .3865. (**I**) Kaplan–Meier curves for OS rate (%) in 13 MF/SS patients with stage IV group (8 in SLFN11-positive group and 5 in SLFN11-negative group), HR, 0.7759; 95% CI, 0.1665–3.614; *P*= .3823. (**J**) Kaplan–Meier curves for OS rate (%) in 13 MF/SS patients with stage IV group (9 in CD30-positive group and 4 in CD30-negative group), HR, 0.7263; 95% CI, 0.1542–3.422; *P*= .8591. N.S., not significant.

This cohort study enrolled 25 Japanese patients with MF and 5 with SS. The mean age at the time of skin biopsy for SLFN11 evaluation was 63.9 years, and the cohort included 20 males and 10 females (Table 1). The distribution of clinical stages was as follows: four patients at stage IA, five at stage IB, seven at stage IIB, one at stage IIIA, four at stage IVA1, eight at stage IVA2, and one at stage IVB (Table 1). Based on the cut-off value at 5%, 19 patients were CD30-positive and 11 were CD30-negative (Fig. 1A–C and Table 1). The mean serum levels of LDH and sIL-2R were 215 U/l and 1029 U/ml, respectively (Table 1). All detailed clinicopathological characteristics are shown in Table 1 and Supplementary Table S1.

**Table 1.** Clinical characteristics of MF/SS patients

Characteristics	Entire cohort (*n* = 30)
Sex, n (%)	
Male	20 (66.7)
Female	10 (33.3)
Age, *y*	
Mean ± SD	63.9 ± 12.3
Clinical diagnosis, *n* (%)	
MF	25 (83.3)
SS	5 (16.7)
Clinical stage, *n* (%)	
Stage IA	4 (13.3)
Stage IB	5 (16.7)
Stage IIB	7 (23.3)
Stage IIIA	1 (3.3)
Stage IVA1	4 (13.3)
Stage IVA2	8 (26.7)
Stage IVB	1 (3.3)
CD30, *n* (%)	
Negative	11 (36.7)
Positive	19 (63.3)
Serum LDH value	
Mean ± SD (normal 115–208 U/l)	215 ± 69.7
Serum sIL-2R value	
Mean ± SD (normal 127–582 U/ml)	1029 ± 1195

Abbreviations: SD, standard deviation; MF, mycosis fungoides; SS, Sezary syndrome; LDH, lactate dehydrogenase; sIL-2R, soluble interleukin-2 receptor.

### SLFN11 expression is positively correlated to CD30 expression in MF/SS

To explore the clinical implications of SLFN11 in MF/SS patients, we assessed SLFN11 expression levels in skin-infiltrating tumor cells using immunohistochemical staining (Fig. 1D, top). Vascular endothelial cells showing a consistent positivity for SLFN11 serve as a positive control in each sample (Fig. 1D, bottom) [41]. We quantified the percentage of SLFN11-positive (SLFN11^+^) cells using image analysis software and set a cut-off value at 10% to classify tumors as SLFN11-positive or SLFN11-negative (Fig. 1E and F). Accordingly, approximately half of MF patients (13/25) and 80% of SS (4/5) patients were classified as SLFN11-positive (Table 2). Univariate analysis revealed no significant association between SLFN11 expression and sex, age (older or younger than 60 years), clinical diagnosis (MF or SS), elevation of serum LDH or sIL-2R values, and TNMB stage (early stage or advanced stage). CD30 expression, however, was significantly associated with SLFN11 expression [odds ratio (OR): 6.899, 95% confidence interval (CI): 1.108–57.875, *P*= .0227] (Table 2). To minimize confounding factors, we performed a multivariate analysis by using four separate models as follows: model 1 (independent variable: sex, age, clinical diagnosis, LDH; dependent variable: SLFN11), model 2 (independent variable: sex, age, clinical diagnosis, sIL-2R; dependent variable: SLFN11), model 3 (independent variable: sex, age, clinical diagnosis, CD30; dependent variable: SLFN11), and model 4 (independent variable: sex, age, clinical diagnosis, TNMB stage; dependent variable: SLFN11), respectively. There was no significant association between SLFN11 expression and other fixed variables (sex, age, and clinical diagnosis). Since the OR, 95% CI, and *P*-value were different in each model, results for fixed variables were omitted in Table 2. In addition, LDH, sIL-2R, and TNMB stage had no association with SLFN11 expression (Table 2). In contrast, CD30 expression remained the only independent significant factor associated with SLFN11 expression (OR: 7.300, 95% CI: 1.0900–48.70, *P*= .0401) (Table 2). We examined the linearity between SLFN11-positive % and CD30-positive %, as well as between SLFN11-positive % and serum IL-2R level. However, we found no significant correlation between them (Fig. 1G), suggesting that SLFN11 positivity, rather than the proportion of SLFN11-positive cells, is associated with CD30 positivity.

**Table 2.** Association between SLFN11 expression and clinicopathological characteristics in MF/SS patients

Characteristics	SLFN11 expression, *n* (%)			Univariate analysis			Multivariate analysis		
	Negative	Positive	Total	OR	95% CI	*P*-value	OR	95% CI	*P*-value
Sex, *n* (%)									
Female	5 (50.0)	5 (50.0)	10 (100)	1.000	0.248–8.967	.705	–	–	–
Male	8 (40.0)	12 (60.0)	20 (100)	1.479					
Age, *n* (%)									
≦60	3 (27.3)	8 (72.7)	11 (100)	1.000	0.0453–2.0727	.259	–	–	–
>60	10 (52.6)	9 (47.4)	19 (100)	0.350					
Clinical diagnosis, *n* (%)									
MF	12 (48.0)	13 (52.0)	25 (100)	1.000	0.294–196.481	.355	–	–	–
SS	1 (20.0)	4 (80.0)	5 (100)	3.549					
Serum LDH value,									
Elevated	3 (42.9)	4 (57.1)	7 (100)	1.000	0.106–6.821	1.000	1.000	0.1260–8.23	.988
Within normal	10 (45.5)	12 (54.5)	22 (100)	0.903			1.020		
Serum sIL-2R value, *n* (%)									
Elevated	5 (31.3)	11 (68.7)	16 (100)	1.000	0.0463–1.661	.144	1.000	0.0633–2.01	.242
Within normal	8 (61.5)	5 (38.5)	13 (100)	0.297			0.357		
CD30, *n* (%)									
Negative	8 (72.7)	3 (27.3)	11 (100)	1.000	1.108–57.875	*.0227	1.000	1.0900–48.70	*.0401
Positive	5 (26.3)	14 (73.7)	19 (100)	6.899			7.300		
TNMB stage, *n* (%)									
Advanced stage (IIB–IVB)	9 (42.9)	12 (57.1)	21 (100)	1.000	0.149–6.218	1.000	1.000	0.2440–7.56	.727
Early stage (IA–IB)	4 (44.4)	5 (55.6)	9 (100)	0.939			1.360		

Abbreviations: MF, mycosis fungoides; SS, Sezary syndrome; LDH, lactate dehydrogenase; sIL-2R, soluble interleukin-2 receptor; OR, odds ratio; CI, confidence interval.

Data for fixed variables (sex, age, and clinical diagnosis) are omitted.

We then analyzed the OS times of MF/SS patients by dividing them into three groups: stage IA–IB (early-stage MF), stage IIB–III (advanced-stage MF), and stage IV (advanced-stage MF and SS). Kaplan–Meier analysis showed that the median OS time of stage IV was significantly shorter than those in the other stages (stage IA–IB was not reached, stage IIB–III was 258 months, and stage IV was 31 months; Fig. 1H). Within the stage IV group, neither SLFN11 nor CD30 expression was associated with OS as determined by the log-rank test (Fig. 1I and J). These findings provide a possibility that SLFN11 and CD30 expressions may be co-regulated, yet they do not directly impact prognosis in MF/SS patients.

### Co-expression of SLFN11 and CD30 in CTCL

**Figure 2.**
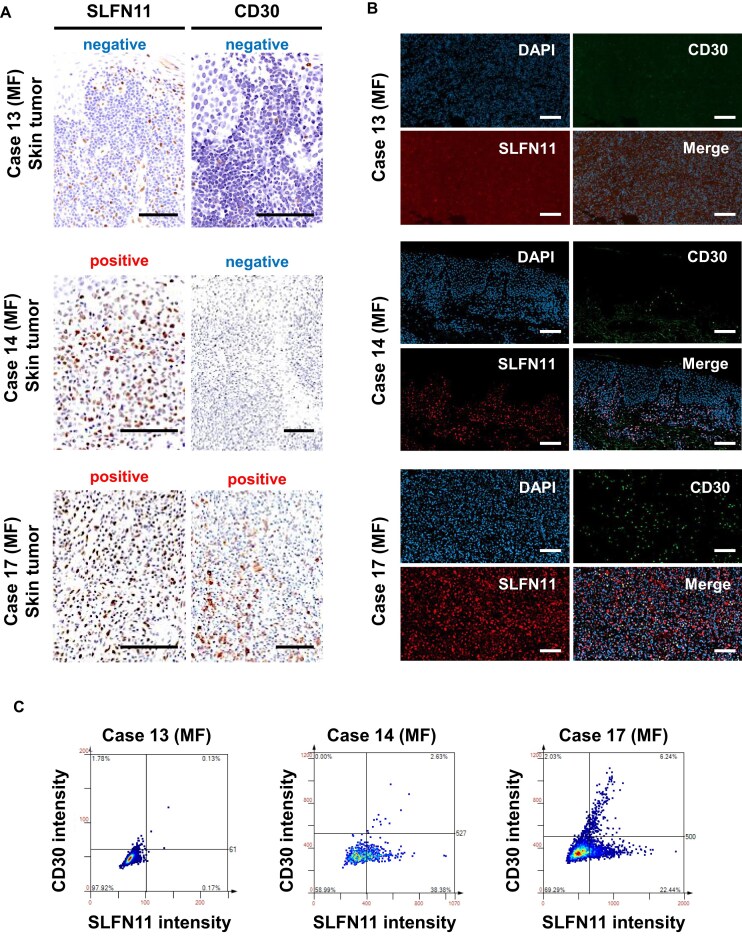
Co-expression of SLFN11 and CD30 in MF tumor cells. (**A**) Representative images of immunohistochemical staining for SLFN11 and CD30 in skin-infiltrating MF tumor cells (original magnification, scale bar indicating 100 μm). (**B**) Representative images of immunofluorescence staining for SLFN11 (red), CD30 (green), and DAPI (blue) corresponding to Fig. 2A (original magnification, scale bar indicating 100 μm). (**C**) An image cytometry analysis by StrataQuest 7 shows the intensity of SLFN11 and CD30 in each cell.

To investigate the possibility of the co-expression of CD30 and SLFN11 in CTCL tumor cells, we further assessed the SLFN11 and CD30 expressions at the single-cell level using immunofluorescence and image cytometry analysis in three patterns of MF samples. In Case 13, both SLFN11 and CD30 were negative by immunohistochemical staining and immunofluorescence staining (Fig. 2A–C). Case 14 was positive for SLFN11 but negative for CD30, while Case 17 was positive for both SLFN11 and CD30, with CD30-positive cells showing predominantly linear correlation with SLFN11 expression (Fig. 2A–C). Although we have not quantified all the samples, Case 17 provided a possibility that CD30 expression may be partly co-regulated with SLFN11 expression.

We then extended the idea of SLFN11 and CD30 co-expression using other CD30-positive lymphoproliferative disorders, including two LyP and five pcALCL, as well as one CD30-positive ATL. SLFN11-positive cases are shown in Supplementary Fig. S1. In cases of CD30-positive LyP and pcALCL available for the single-cell level analysis, the immunofluorescence and image cytometry demonstrated concomitant expression of CD30 and SLFN11 overall (Supplementary Fig. S2A and B). To further examine the co-expression of SLFN11 and CD30 in a broad range of cancer cell lines, we analyzed the GDSC cell line database using the CellMinerCDB website [45]. Although there was no correlation between SLFN11 and CD30 (TNFRSF8) across all the cancer cell lines (Supplementary Fig. S3A), a significant positive correlation was observed, exclusively in lymphoma cell lines (Supplementary Fig. S3B). These findings suggest a preferential co-expression of SLFN11 and CD30 in CD30-positive CTCLs, a relationship that also extends to other CD30-positive lymphoid malignancies, particularly within lymphoma cell lines.

### Activation of normal T cells induces CD30 and SLFN11 co-expression

**Figure 3.**
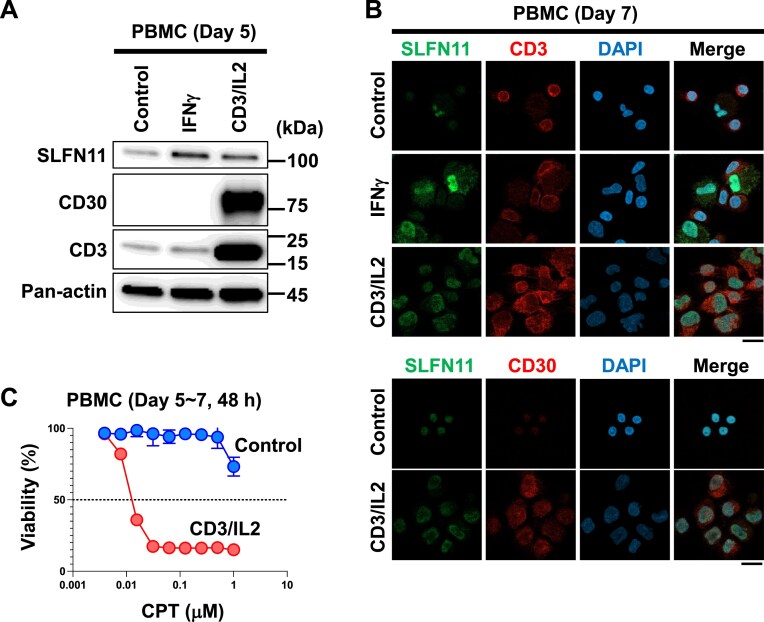
Activation via T-cell receptor/IL-2R induces the functional SLFN11 expression in normal T cells. (**A**) Immunoblotting showing SLFN11, CD30, and CD3 expressions in PBMCs with or without IFN-γ or CD3/CD28/IL-2 stimulation for 5 days. Actin was used as a loading control. (**B**) Representative confocal microscopy images for SLFN11 (green), CD3 (red), and DAPI (blue) (top panel) or SLFN11 (green), CD30 (red), and DAPI (blue) (bottom panel) in PBMCs 7 days after each activation (original magnification, scale bar indicating 10 μm). (**C**) Viability curves to various concentrations of TOP1 inhibitor CPT in PBMCs with or without CD3/CD28/IL-2 stimulation for 5–7 days. Viability was examined by ATP assay 48 h after the CPT treatments. Representative results in triplicate from two independent experiments are shown as mean ± SD.

Since the normal counterparts of MF/SS are αβ T cells, we assessed the co-expression of SLFN11 and CD30 in normal human PBMCs. In general, IFNγ activates macrophages, while CD3, CD28, and IL-2 activate T cells. As macrophages in normal conditions express high SLFN11 [41], IFN-γ upregulated the SLFN11 expression in cells displaying macrophage-like morphology (Fig. 3A and B). Conversely, PBMCs stimulated with CD3/CD28/IL-2 upregulated SLFN11, CD3, and CD30 expressions, as observed in immunoblots (Fig. 3A). In immunofluorescence staining images, SLFN11-positive cells were also positive for CD3 and CD30 (Fig. 3B). These results indicate that T-cell activation via T-cell receptor with IL-2R triggers the simultaneous expression of SLFN11 and CD30.

To examine whether the induction of SLFN11 affects sensitivity to DNA-damaging agents, we measured the viability of untreated or CD3/CD28/IL-2-stimulated PBMCs under CPT, a topoisomerase I inhibitor. CD3/CD28/IL-2-stimulated PBMCs demonstrated increased sensitivity to CPT in a dose-dependent manner compared to untreated PBMCs (Fig. 3C). These results suggest that normal CD3-positive T cells can readily express SLFN11 and CD30 in response to CD3/CD28/IL-2 stimulation, acquiring hypersensitivity to DNA-damaging agents.

### SLFN11 determines sensitivity to DNA-damaging agents in CTCL cell lines

**Figure 4.**
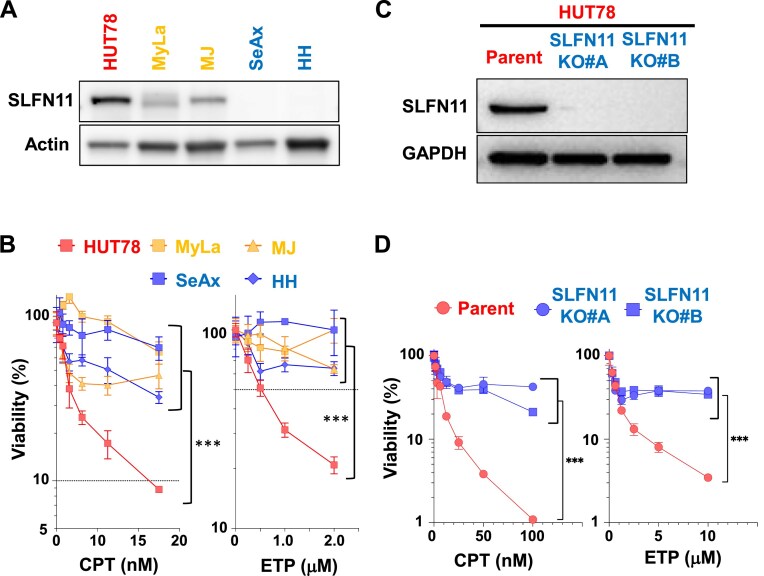
SLFN11 expression determines sensitivity to DNA-targeting agents, TOP1 and TOP2 inhibitors in CTCL cell lines. (**A**) Immunoblotting showing SLFN11 expression in the indicated CTCL cell lines. Actin was used as a loading control. (**B**) Viability curves of the indicated CTCL cell lines to various concentrations of TOP1 inhibitor (CPT) or TOP2 inhibitor (ETP). (**C**) Immunoblotting showing SLFN11 expression in the HUT78 parental and SLFN11 knockout (SLFN11-KO#A and #B) cells. GAPDH was used as a loading control. (**D**) Viability curves of the HUT78 parental and SLFN11 KO cells to CPT and ETP. (B, D) Viability was examined by ATP assay 72 h after the drug treatments. Representative results in triplicate from two or three independent experiments are shown as mean ± SD. ***P< .001 by one-way ANOVA followed by Dunnett’s multiple-comparisons test.

To investigate the role of SLFN11 in CTCL cells, we examined its expression across five CTCL cell lines: HUT78, MyLa, MJ, SeAx, and HH. High levels of SLFN11 expression were detected in HUT78, with weaker expression observed in MyLa and MJ. No SLFN11 expression was detected in SeAx or HH (Fig. 4A). HUT78 exhibited the highest sensitivity to CPT and a topoisomerase II inhibitor ETP among the CTCL cell lines (Fig. 4B). To examine the contribution of SLFN11 to the highest sensitivity of HUT78, we generated two SLFN11 knockout cells (SLFN11 KO#A, SLFN11 KO#B) in the HUT78 cell using CRISPR–Cas9 system and two different guide RNAs (Fig. 4C). SLFN11 KO cells significantly developed resistance to CPT and ETP compared to the parental cells (Fig. 4D). These findings suggest that SLFN11 expression is a critical determinant for sensitivity to DNA-targeting agents in CTCL.

### Inconsistent SLFN11 induction and anticancer activity of (pre)clinical HDAC inhibitors in CTCL

**Figure 5.**
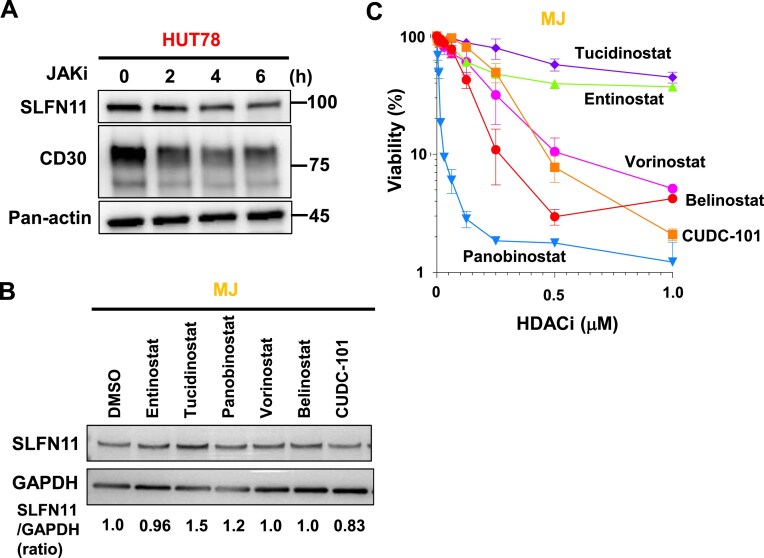
JAK pathway activation and epigenetic suppression regulate SLFN11 expression in CTCL cell lines. (**A**) Immunoblotting showing SLFN11 and CD30 expressions in HUT78 cells treated with pan-JAK inhibitor, cerdulatinib (40 μM), for the indicated time. Actin was used as a loading control. A representative result of two independent experiments is shown. (**B**) Immunoblotting showing SLFN11 expression in MJ cells treated with the indicated HDAC inhibitors (10 μM each) for 16 h. GAPDH was used as a loading control. The relative ratio of SLFN11/GAPDH in each HDAC inhibitor-treated MJ cell is indicated at the bottom. A representative result of three independent experiments is shown. (**C**) Viability curves of the MJ cells to the indicated HDAC inhibitors. Viability was examined by ATP assay 72 h after the drug treatments. Representative results in triplicate from two independent experiments are shown as mean ± SD.

To further understand the co-regulation of SLFN11 and CD30 in CTCL, we investigated their regulatory mechanisms. Previously, we reported that gain-of-function mutations in Janus kinase (JAK) contribute to upregulation of SLFN11 in human leukemia cells [46]. As noted in those cells, a clinical JAK inhibitor, cerdulatinib, suppressed the SLFN11 expression also in HUT78 cells (Fig. 5A). Interestingly, CD30 expression was also suppressed by the JAK inhibitor (Fig. 5A). These findings indicate that the JAK pathway may contribute the co-expression of SLFN11 and CD30 in CTCL tumor cells.

Finally, we examined the utility of HDAC inhibitors to activate SLFN11, since SLFN11 expression is epigenetically suppressed. A couple of HDAC inhibitors have been applied to the therapeutic option for CTCL, although their response rates vary [47]. Hence, we tested six preclinical or clinical HDAC inhibitors to evaluate their ability to increase SLFN11 expression in MJ cells and MyLa cells, which exhibit weak SLFN11 expression (Fig. 4A). For MJ cells, tucidinostat was the most effective inducer of SLFN11 expression (Fig. 5B). At the same time, none of them induced or some of them even reduced SLFN11 expression in MyLa cells (Supplementary Fig. S4), which complicates the use of HDAC inhibitors intended to induce SLFN11. Moreover, the toxicity profiles of these HDAC inhibitors varied greatly in MJ cells, with panobinostat displaying the highest toxicity and tucidinostat the lowest (Fig. 5C). These results suggest that HDAC inhibitors could be selected based on therapeutic goals: for example, tucidinostat may be preferred for SLFN11 induction, while panobinostat may be used for its potent cytotoxic effect.

## Discussion

Our cohort study unveiled that CD30 expression positively correlates with SLFN11 expression in CTCL malignant T cells. The causality was validated by simultaneous activation of SLFN11 and CD30 in normal T cells via T-cell receptor/IL-2R stimuli and by simultaneous suppression of SLFN11 and CD30 by JAK inhibition. Consistent with the cumulated evidence in other cancers, SLFN11 was a major determinant of sensitivity to ETP and CPT in tested CTCL cell lines. Hence, we propose a rationale for developing SLFN11-dependent drugs such as TOP1 and TOP2 inhibitors as payloads of anti-CD30 monoclonal antibody-conjugated therapy.

SLFN11 expression serves as a favorable prognostic marker in many types of cancers when patients are treated with DNA-damaging agents containing chemotherapy. In our cohort, the median OS for stage IV was 31 months, which was similar to the previous report showing 47.5 months for stage IVA and 33.3 months for stage IVB [7]. All the stage IV patients, except for one SS patient, were treated with at least one systemic therapy in this study (Supplementary Table S1). However, neither SLFN11 nor CD30 expression had a significant impact on the prognosis of stage IV patients. When we evaluated the relationship between SLFN11 expression and the presence or absence of systemic therapies, including before and after the final staging, no statistical significance was found (data not shown). In addition, there was no association between SLFN11 expression and the number of administered systemic therapies (one or more than one; data not shown). A limited number of cases were enrolled in this study, and the treatments received varied widely in each case. Therefore, we were unable to conclude that SLFN11 is a predictive biomarker for prognosis or sensitivity to DNA-damaging anticancer agents in CTCL patients in this cohort.

In this study, we set the cut-off values of SLFN11 and CD30 to be defined as 10% and 5%, respectively. Referring to the previous reports, the clinical cut-off value of SLFN11 ranged from 5% to 30% [31,, 32, 36]. Thus, we set a cut-off value at 10% to classify tumors as SLFN11-positive or SLFN11-negative in our study. On the other hand, the criteria of the clinical cut-off value of CD30 positivity remain unclear. A phase II clinical trial of brentuximab vedotin demonstrated that lower CD30 expression (<5%) had a lower likelihood of global response than higher CD30 expression (≧5%) [48]. Based on this data, CD30 positivity was defined as ≧5% in this study. Among transformed MF, characterized by the presence of >25% large cell transformed atypical cells in skin-infiltrating cells, CD30 expression is detected in approximately half of the patients. Although large-cell transformation is an independent prognostic factor for worse survival, the relationship between CD30 expression and prognosis in MF/SS remains controversial. Expression of CD30 in the tumor stage of non-transformed MF has been identified as a poor prognostic marker [7]. In contrast, a recent systematic review and meta-analysis showed that the expression of CD30 in transformed MF was a significant favorable prognostic factor, while the mechanisms have not been elucidated [49].

T-cell receptor stimulation promotes IL-2 production in T cells. Subsequently, the binding of IL-2 to the IL-2R complex activates the signaling pathways, including STAT1/3/5, PI3K/AKT, and RAF/MEK/ERK via JAK1/3 [50]. We previously reported that gain-of-function mutations of JAK activate SLFN11 expression in human acute leukemia cells [46]. The gain-of-function mutations of JAK have also been reported in CTCL, especially in advanced-stage MF/SS [51–54]. Interestingly, 50% of cutaneous CD30-positive lymphoproliferative disorders, the second common form of CTCL (LyP and pcALCL), harbor a constitutively activated JAK signal pathway [55]. Hence, the CD30-positive background may affect the co-expression of SLFN11 via JAK signal activation. Since CD30 is not a transcription factor and SLFN11’s potential transcriptional role remains unclear, direct regulation of one by the other appears unlikely. Otherwise, our data using normal healthy PBMCs with CD3/CD28/IL-2 stimulation support the notion that the T-cell receptor/IL-2R/JAK signaling pathway may directly regulate the expression of both SLFN11 and CD30 in CD3-positive T cells. We have also acknowledged that other regulatory pathways may be involved.

We revealed that SLFN11 is functional in a CTCL cell line, HUT78, and enhances sensitivity to CPT and ETP (Fig. 4). Our results suggest that analyzing SLFN11 expression could be beneficial for selecting drugs in CTCL. Concerning pathobiological relationships between primary patient samples and CTCL cell lines used in this study, gene expression analysis by a previous study demonstrated that MyLa cells represent MF, HH present CD30-positive MF and/or ALCL, and SeAx and Hut78 cells represent SS, respectively [42]. MJ cells showed a similar expression profile of SS but represent a leukemic form of ATL [42]. Thus, our *in vitro* data may compensate the hypothesis that SLFN11 will be a predictive biomarker for sensitivity to DNA-damaging agents in CD30-positive CTCL patients, at least leukemic MF or SS patients.

Currently, brentuximab vedotin is an antibody–drug conjugate (ADC) that exerts its therapeutic effect by binding CD30 and inhibiting microtubule polymerization with MMAE as a payload. A phase 3, open-label randomized controlled trial comparing the brentuximab vedotin group with bexarotene or methotrexate group showed a significantly superior response rate to MF, with 65% in the brentuximab vedotin group and 16% in the bexarotene or methotrexate group [56]. Because SLFN11 does not augment sensitivity to microtube polymerization inhibitors, CD30-specific antibody–DNA-damaging agent conjugates may be reasonable for CD30 and SLFN11 double-positive CTCL. ETP, an SLFN11-dependent DNA-damaging agent, has been applied to recurrent and progressive MF patients with a response rate of 69% [57]. Therefore, we propose to develop an ADC combining anti-CD30 antibody with ETP as a new therapeutic drug.

Among HDAC inhibitors, the Food and Drug Administration approved vorinostat and romidepsin for CTCL patients, but their response rates are limited to around 30% [58, 59]. Therefore, combination therapies with various anticancer drugs such as bexarotene [60], azacytidine [61], lenalidomide [62], PI3K inhibitor [63], ruxolitinib (JAK1/2 inhibitor) [64], BET inhibitors [65, 66], and BCL2 inhibitor (venetoclax) [67] have been performed in the clinic or tested in *in vitro* systems. Our study revealed that HDAC inhibitors greatly vary in cytotoxicity and slightly vary in the reactivation of SLFN11 expression in MJ cells. For example, panobinostat showed strong cytotoxicity while showing potent SLFN11 reactivation, suggesting a candidate for a single agent targeting CTCL. In contrast, tucidinostat—approved in Japan for recurrent or progressive ATL and peripheral T-cell lymphoma—exhibited lower cytotoxicity toward MJ cells but induced SLFN11 expression to a measurable extent. We investigated whether combining an HDAC inhibitor (tucidinostat) with CPT or ETP could enhance the cytotoxic effect on SLFN11 weakly expressing MJ cells. However, we cannot obtain positive results (data not shown). Since HDAC inhibitors have cell toxicity for CTCL tumor cells, it would be difficult to make a sharp contrast between the combination of HDAC inhibitor + DNA-damaging agent and DNA-damaging agent alone. If the tumor cells were strongly resistant to HDAC inhibitors, a synergistic effect of combination therapy on CTCL could be expected.

In conclusion, we shed light on a new therapeutic strategy for CTCL from the aspect of SLFN11. ADCs combining anti-CD30 antibodies with DNA-targeted reagents will be expected to respond well to CD30 and SLFN11 double-positive CTCL patients. More extensive clinical data should be required to draw convincing conclusions in our study.

## Supplementary Material

zcaf037_Supplemental_Files

## Data Availability

The data underlying this article will be shared on reasonable request to the corresponding authors.

## References

[1] Board TWCoTE WHO Classification of Tumours. 2024; 5th edn Lyon IARC Press.

[2] Fujii K, Hamada T, Shimauchi T et al. Cutaneous lymphoma in Japan, 2012–2017: a nationwide study. J Dermatol Sci. 2020; 97:187–93. 10.1016/j.jdermsci.2020.01.010. 32033869

[3] Olsen EA, Whittaker S, Willemze R et al. Primary cutaneous lymphoma: recommendations for clinical trial design and staging update from the ISCL, USCLC, and EORTC. Blood. 2022; 140:419–37. 10.1182/blood.2021012057. 34758074 PMC9353153

[4] Agar NS, Wedgeworth E, Crichton S et al. Survival outcomes and prognostic factors in mycosis fungoides/Sezary syndrome: validation of the revised International Society for Cutaneous Lymphomas/European Organisation for Research and Treatment of Cancer staging proposal. J Clin Oncol. 2010; 28:4730–9. 10.1200/JCO.2009.27.7665. 20855822

[5] Talpur R, Singh L, Daulat S et al. Long-term outcomes of 1,263 patients with mycosis fungoides and Sezary syndrome from 1982 to 2009. Clin Cancer Res. 2012; 18:5051–60. 10.1158/1078-0432.CCR-12-0604. 22850569 PMC3857608

[6] Quaglino P, Pimpinelli N, Berti E et al. Time course, clinical pathways, and long-term hazards risk trends of disease progression in patients with classic mycosis fungoides: a multicenter, retrospective follow-up study from the Italian Group of Cutaneous Lymphomas. Cancer. 2012; 118:5830–9. 10.1002/cncr.27627. 22674564

[7] Scarisbrick JJ, Prince HM, Vermeer MH et al. Cutaneous Lymphoma International Consortium study of outcome in advanced stages of mycosis fungoides and Sezary syndrome: effect of specific prognostic markers on survival and development of a prognostic model. J Clin Oncol. 2015; 33:3766–73. 10.1200/JCO.2015.61.7142. 26438120 PMC4979132

[8] Gilson D, Whittaker SJ, Child FJ et al. British Association of Dermatologists and U.K. Cutaneous Lymphoma Group guidelines for the management of primary cutaneous lymphomas 2018. Br J Dermatol. 2019; 180:496–526. 10.1111/bjd.17240. 30561020

[9] Latzka J, Assaf C, Bagot M et al. EORTC consensus recommendations for the treatment of mycosis fungoides/Sezary syndrome—update 2023. Eur J Cancer. 2023; 195:113343 10.1016/j.ejca.2023.113343. 37890355

[10] Willemze R, Hodak E, Zinzani PL et al. Primary cutaneous lymphomas: ESMO Clinical Practice Guidelines for diagnosis, treatment and follow-up. Ann Oncol. 2018; 29:iv30–40. 29878045 10.1093/annonc/mdy133

[11] Zoppoli G, Regairaz M, Leo E et al. Putative DNA/RNA helicase Schlafen-11 (SLFN11) sensitizes cancer cells to DNA-damaging agents. Proc Natl Acad Sci USA. 2012; 109:15030–5. 22927417 10.1073/pnas.1205943109 PMC3443151

[12] Barretina J, Caponigro G, Stransky N et al. The Cancer Cell Line Encyclopedia enables predictive modelling of anticancer drug sensitivity. Nature. 2012; 483:603–7. 22460905 10.1038/nature11003 PMC3320027

[13] Jo U, Murai Y, Takebe N et al. Precision Oncology with drugs targeting the replication stress, ATR, and Schlafen 11. Cancers. 2021; 13:4601. 34572827 10.3390/cancers13184601 PMC8465591

[14] Murai J, Thomas A, Miettinen M et al. Schlafen 11 (SLFN11), a restriction factor for replicative stress induced by DNA-targeting anti-cancer therapies. Pharmacol Ther. 2019; 201:94–102. 10.1016/j.pharmthera.2019.05.009. 31128155 PMC6708787

[15] Marzi L, Szabova L, Gordon M et al. The indenoisoquinoline TOP1 inhibitors selectively target homologous recombination-deficient and Schlafen 11-positive cancer cells and synergize with olaparib. Clin Cancer Res. 2019; 25:6206–16. 10.1158/1078-0432.CCR-19-0419. 31409613 PMC6801079

[16] Onji H, Tate S, Sakaue T et al. Schlafen 11 further sensitizes BRCA-deficient cells to PARP inhibitors through single-strand DNA gap accumulation behind replication forks. Oncogene. 2024; 43:2475–89. 10.1038/s41388-024-03094-1. 38961202 PMC11315672

[17] Li M, Kao E, Malone D et al. DNA damage-induced cell death relies on SLFN11-dependent cleavage of distinct type II tRNAs. Nat Struct Mol Biol. 2018; 25:1047–58. 10.1038/s41594-018-0142-5. 30374083 PMC6579113

[18] Malone D, Lardelli RM, Li M et al. Dephosphorylation activates the interferon-stimulated Schlafen family member 11 in the DNA damage response. J Biol Chem. 2019; 294:14674–85. 10.1074/jbc.RA118.006588. 31395656 PMC6779438

[19] Li M, Kao E, Gao X et al. Codon-usage-based inhibition of HIV protein synthesis by human Schlafen 11. Nature. 2012; 491:125–8. 10.1038/nature11433. 23000900 PMC3705913

[20] Boon NJ, Oliveira RA, Korner PR et al. DNA damage induces p53-independent apoptosis through ribosome stalling. Science. 2024; 384:785–92. 10.1126/science.adh7950. 38753784

[21] Fujiwara K, Maekawa M, Iimori Y et al. The crucial role of single-stranded DNA binding in enhancing sensitivity to DNA-damaging agents for Schlafen 11 and Schlafen 13. iScience. 2023; 26:108529. 38125019 10.1016/j.isci.2023.108529 PMC10730379

[22] Murai J, Tang SW, Leo E et al. SLFN11 blocks stressed replication forks independently of ATR. Mol Cell. 2018; 69:371–84. 29395061 10.1016/j.molcel.2018.01.012 PMC5802881

[23] Mu Y, Lou J, Srivastava M et al. SLFN11 inhibits checkpoint maintenance and homologous recombination repair. EMBO Rep. 2016; 17:94–109. 10.15252/embr.201540964. 26658330 PMC4718411

[24] Metzner FJ, Wenzl SJ, Kugler M et al. Mechanistic understanding of human SLFN11. Nat Commun. 2022; 13:5464 10.1038/s41467-022-33123-0. 36115853 PMC9482658

[25] Murai J, Ceribelli M, Fu H et al. Schlafen 11 (SLFN11) kills cancer cells undergoing unscheduled re-replication. Mol Cancer Ther. 2023; 22:985–95. 10.1158/1535-7163.MCT-22-0552. 37216280 PMC10524552

[26] Ogawa A, Izumikawa K, Tate S et al. SLFN11-mediated ribosome biogenesis impairment induces TP53-independent apoptosis. Mol Cell. 2025; 85:894–912. 10.1016/j.molcel.2025.01.008. 39909041 PMC11890970

[27] Coussy F, El-Botty R, Chateau-Joubert S et al. BRCAness, SLFN11, and RB1 loss predict response to topoisomerase I inhibitors in triple-negative breast cancers. Sci Transl Med. 2020; 12:eaax2625 10.1126/scitranslmed.aax2625. 32075943 PMC8662740

[28] Willis SE, Winkler C, Roudier MP et al. Retrospective analysis of Schlafen11 (SLFN11) to predict the outcomes to therapies affecting the DNA damage response. Br J Cancer. 2021; 125:1666–76. 10.1038/s41416-021-01560-1. 34663950 PMC8651811

[29] Winkler C, King M, Berthe J et al. SLFN11 captures cancer-immunity interactions associated with platinum sensitivity in high-grade serous ovarian cancer. JCI Insight. 2021; 6:e146098 10.1172/jci.insight.146098. 34549724 PMC8492341

[30] Akashi H, Yachida N, Ueda H et al. SLFN11 is a BRCA independent biomarker for the response to platinum-based chemotherapy in high-grade serous ovarian cancer and clear cell ovarian carcinoma. Mol Cancer Ther. 2023; 23:106–116. 10.1158/1535-7163.MCT-23-0257 37717249

[31] Takashima T, Taniyama D, Sakamoto N et al. Schlafen 11 predicts response to platinum-based chemotherapy in gastric cancers. Br J Cancer. 2021; 125:65–77. 10.1038/s41416-021-01364-3. 33785877 PMC8257722

[32] Taniyama D, Sakamoto N, Takashima T et al. Prognostic impact of Schlafen 11 in bladder cancer patients treated with platinum-based chemotherapy. Cancer Sci. 2022; 113:784–95. 10.1111/cas.15207. 34808009 PMC8819307

[33] Lok BH, Gardner EE, Schneeberger VE et al. PARP inhibitor activity correlates with SLFN11 expression and demonstrates synergy with temozolomide in small cell lung cancer. Clin Cancer Res. 2017; 23:523–35. 10.1158/1078-0432.CCR-16-1040. 27440269 PMC5241177

[34] Thomas A, Takahashi N, Rajapakse VN et al. Therapeutic targeting of ATR yields durable regressions in small cell lung cancers with high replication stress. Cancer Cell. 2021; 39:566–79. 10.1016/j.ccell.2021.02.014. 33848478 PMC8048383

[35] Kagami T, Yamade M, Suzuki T et al. The first evidence for SLFN11 expression as an independent prognostic factor for patients with esophageal cancer after chemoradiotherapy. BMC Cancer. 2020; 20:1123 10.1186/s12885-020-07574-x. 33218331 PMC7678160

[36] Hamada S, Kano S, Murai J et al. Schlafen family member 11 indicates favorable prognosis of patients with head and neck cancer following platinum-based chemoradiotherapy. Front Oncol. 2022; 12:978875 10.3389/fonc.2022.978875. 36741698 PMC9892834

[37] Conteduca V, Ku SY, Puca L et al. SLFN11 expression in advanced prostate cancer and response to platinum-based chemotherapy. Mol Cancer Ther. 2020; 19:1157–64. 10.1158/1535-7163.MCT-19-0926. 32127465 PMC7440143

[38] Nakata S, Murai J, Okada M et al. Epigenetic upregulation of Schlafen11 renders WNT- and SHH-activated medulloblastomas sensitive to cisplatin. Neuro Oncol. 2023; 25:899–912. 10.1093/neuonc/noac243. 36273330 PMC10158119

[39] Moribe F, Nishikori M, Takashima T et al. Epigenetic suppression of SLFN11 in germinal center B-cells during B-cell development. PLoS One. 2021; 16:e0237554 10.1371/journal.pone.0237554. 33513156 PMC7846023

[40] Tang SW, Thomas A, Murai J et al. Overcoming resistance to DNA-targeted agents by epigenetic activation of Schlafen 11 (SLFN11) expression with class I histone deacetylase inhibitors. Clin Cancer Res. 2018; 24:1944–53. 10.1158/1078-0432.CCR-17-0443. 29391350 PMC5899656

[41] Takashima T, Sakamoto N, Murai J et al. Immunohistochemical analysis of SLFN11 expression uncovers potential non-responders to DNA-damaging agents overlooked by tissue RNA-seq. Virchows Arch. 2021; 478:569–79. 10.1007/s00428-020-02840-6. 32474729 PMC9175511

[42] Netchiporouk E, Gantchev J, Tsang M et al. Analysis of CTCL cell lines reveals important differences between mycosis fungoides/Sezary syndrome vs. HTLV-1(+) leukemic cell lines. Oncotarget. 2017; 8:95981–98. 10.18632/oncotarget.21619. 29221181 PMC5707075

[43] Murai J, Feng Y, Yu GK et al. Resistance to PARP inhibitors by SLFN11 inactivation can be overcome by ATR inhibition. Oncotarget. 2016; 7:76534–50. 10.18632/oncotarget.12266. 27708213 PMC5340226

[44] Kanda Y Investigation of the freely available easy-to-use software ‘EZR’ for medical statistics. Bone Marrow Transplant. 2013; 48:452–8. 10.1038/bmt.2012.244. 23208313 PMC3590441

[45] Luna A, Elloumi F, Varma S et al. CellMiner Cross-Database (CellMinerCDB) version 1.2: exploration of patient-derived cancer cell line pharmacogenomics. Nucleic Acids Res. 2021; 49:D1083–93. 10.1093/nar/gkaa968. 33196823 PMC7779001

[46] Murai Y, Jo U, Murai J et al. Schlafen 11 expression in human acute leukemia cells with gain-of-function mutations in the interferon-JAK signaling pathway. iScience. 2021; 24:103173 10.1016/j.isci.2021.103173. 34693224 PMC8517841

[47] Jimura N, Fujii K, Qiao Z et al. Kinome profiling analysis identified Src pathway as a novel therapeutic target in combination with histone deacetylase inhibitors for cutaneous T-cell lymphoma. J Dermatol Sci. 2021; 101:194–201. 10.1016/j.jdermsci.2021.01.004. 33531202

[48] Kim YH, Tavallaee M, Sundram U et al. Phase II investigator-initiated study of brentuximab vedotin in mycosis fungoides and Sezary syndrome with variable CD30 expression level: a multi-institution collaborative project. J Clin Oncol. 2015; 33:3750–8. 10.1200/JCO.2014.60.3969. 26195720 PMC5089160

[49] Travaglino A, Russo D, Varricchio S et al. Prognostic significance of CD30 in transformed mycosis fungoides. Am J Clin Pathol. 2021; 156:350–5. 10.1093/ajcp/aqaa261. 33769436

[50] Carbone F, Russo C, Colamatteo A et al. Cellular and molecular signaling towards T cell immunological self-tolerance. J Biol Chem. 2024; 300:107134 10.1016/j.jbc.2024.107134. 38432631 PMC10981134

[51] Kiel MJ, Sahasrabuddhe AA, Rolland DCM et al. Genomic analyses reveal recurrent mutations in epigenetic modifiers and the JAK-STAT pathway in Sezary syndrome. Nat Commun. 2015; 6:8470 10.1038/ncomms9470. 26415585 PMC4598843

[52] McGirt LY, Jia P, Baerenwald DA et al. Whole-genome sequencing reveals oncogenic mutations in mycosis fungoides. Blood. 2015; 126:508–19. 10.1182/blood-2014-11-611194. 26082451 PMC4513251

[53] Perez C, Gonzalez-Rincon J, Onaindia A et al. Mutated JAK kinases and deregulated STAT activity are potential therapeutic targets in cutaneous T-cell lymphoma. Haematologica. 2015; 100:e450–3. 10.3324/haematol.2015.132837. 26294736 PMC4825308

[54] Perez C, Mondejar R, Garcia-Diaz N et al. Advanced-stage mycosis fungoides: role of the signal transducer and activator of transcription 3, nuclear factor-kappaB and nuclear factor of activated T cells pathways. Br J Dermatol. 2020; 182:147–55. 10.1111/bjd.18098. 31049933

[55] Maurus K, Appenzeller S, Roth S et al. Recurrent oncogenic JAK and STAT alterations in cutaneous CD30-positive lymphoproliferative disorders. J Invest Dermatol. 2020; 140:2023–31. 10.1016/j.jid.2020.02.019. 32147503

[56] Prince HM, Kim YH, Horwitz SM et al. Brentuximab vedotin or physician’s choice in CD30-positive cutaneous T-cell lymphoma (ALCANZA): an international, open-label, randomised, phase 3, multicentre trial. Lancet. 2017; 390:555–66. 10.1016/S0140-6736(17)31266-7. 28600132

[57] Purnak S, Azar J, Mark LA Etoposide as a single agent in the treatment of mycosis fungoides: a retrospective analysis. Dermatol Ther. 2018; 31:e12586 10.1111/dth.12586. 29316111

[58] Olsen EA, Kim YH, Kuzel TM et al. Phase IIb multicenter trial of vorinostat in patients with persistent, progressive, or treatment refractory cutaneous T-cell lymphoma. J Clin Oncol. 2007; 25:3109–15. 10.1200/JCO.2006.10.2434. 17577020

[59] Whittaker SJ, Demierre MF, Kim EJ et al. Final results from a multicenter, international, pivotal study of romidepsin in refractory cutaneous T-cell lymphoma. J Clin Oncol. 2010; 28:4485–91. 10.1200/JCO.2010.28.9066. 20697094

[60] Dummer R, Beyer M, Hymes K et al. Vorinostat combined with bexarotene for treatment of cutaneous T-cell lymphoma: *in vitro* and phase I clinical evidence supporting augmentation of retinoic acid receptor/retinoid X receptor activation by histone deacetylase inhibition. Leuk Lymphoma. 2012; 53:1501–8. 10.3109/10428194.2012.656625. 22239668

[61] Rozati S, Cheng PF, Widmer DS et al. Romidepsin and azacitidine synergize in their epigenetic modulatory effects to induce apoptosis in CTCL. Clin Cancer Res. 2016; 22:2020–31. 10.1158/1078-0432.CCR-15-1435. 26660520

[62] Cosenza M, Civallero M, Fiorcari S et al. The histone deacetylase inhibitor romidepsin synergizes with lenalidomide and enhances tumor cell death in T-cell lymphoma cell lines. Cancer Biol Ther. 2016; 17:1094–106. 10.1080/15384047.2016.1219820. 27657380 PMC5079402

[63] Wozniak MB, Villuendas R, Bischoff JR et al. Vorinostat interferes with the signaling transduction pathway of T-cell receptor and synergizes with phosphoinositide-3 kinase inhibitors in cutaneous T-cell lymphoma. Haematologica. 2010; 95:613–21. 10.3324/haematol.2009.013870. 20133897 PMC2857191

[64] Karagianni F, Piperi C, Mpakou V et al. Ruxolitinib with resminostat exert synergistic antitumor effects in Cutaneous T-cell Lymphoma. PLoS One. 2021; 16:e0248298 10.1371/journal.pone.0248298. 33705488 PMC7951910

[65] Yumeen S, Mirza FN, Lewis JM et al. JAK inhibition synergistically potentiates BCL2, BET, HDAC, and proteasome inhibition in advanced CTCL. Blood Adv. 2020; 4:2213–26. 10.1182/bloodadvances.2020001756. 32437546 PMC7252559

[66] Zhao L, Okhovat JP, Hong EK et al. Preclinical studies support combined inhibition of BET family proteins and histone deacetylases as epigenetic therapy for cutaneous T-Cell lymphoma. Neoplasia. 2019; 21:82–92. 10.1016/j.neo.2018.11.006. 30529073 PMC6280696

[67] Cyrenne BM, Lewis JM, Weed JG et al. Synergy of BCL2 and histone deacetylase inhibition against leukemic cells from cutaneous T-cell lymphoma patients. Blood. 2017; 130:2073–83. 10.1182/blood-2017-06-792150. 28972015 PMC5680613

