# Supplementary material for: Co-expression of CD30 and SLFN11 serves as a dual biomarker for the treatment of cutaneous T-cell lymphoma
Source: NAR Cancer. 2025 Oct 7;7(4):zcaf037. doi: 10.1093/narcan/zcaf037 (PMC12501776; doi:10.1093/narcan/zcaf037)

Supplemental Figure S1

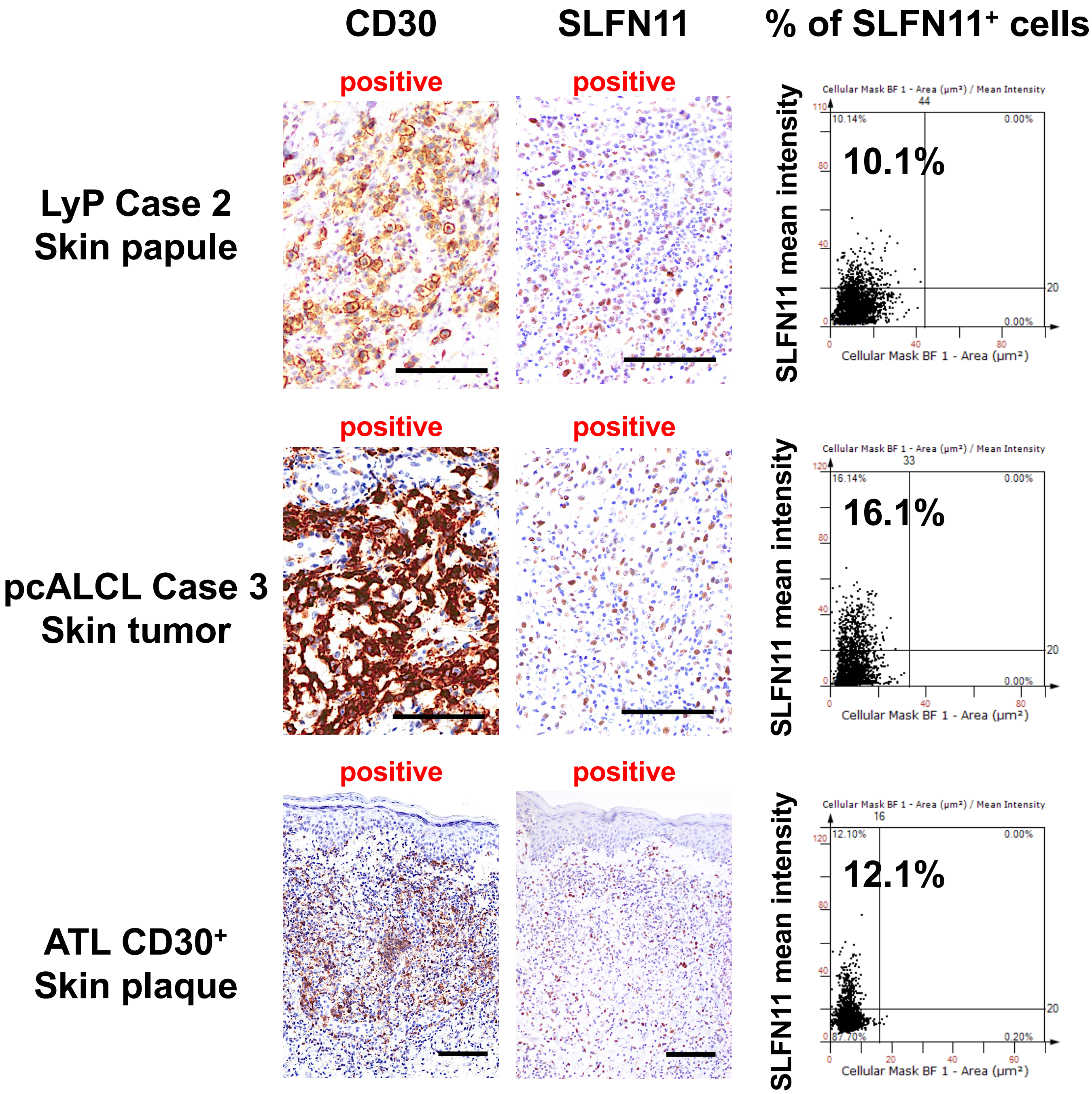

Supplemental Figure S2

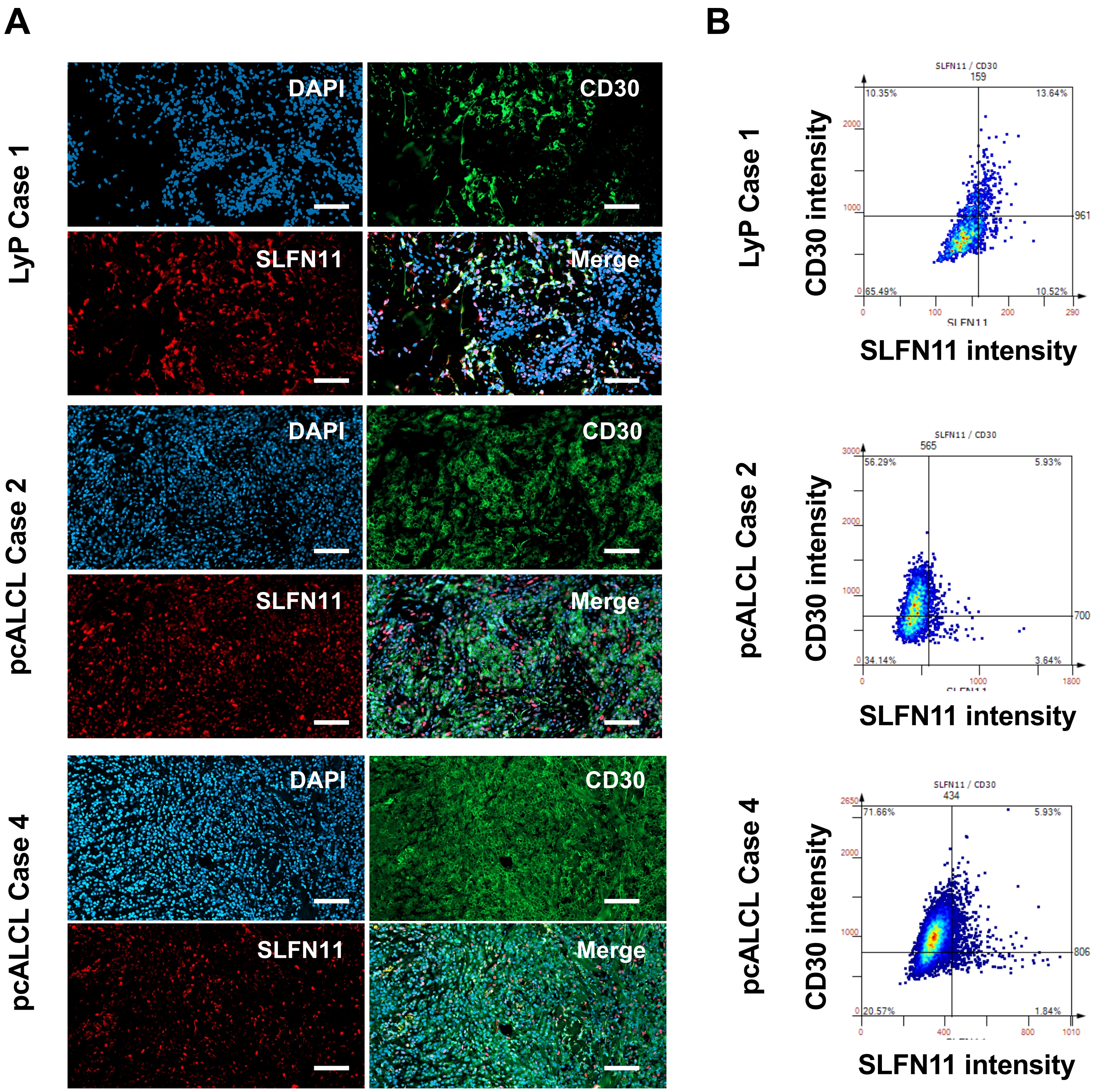

Supplemental Figure S3

A

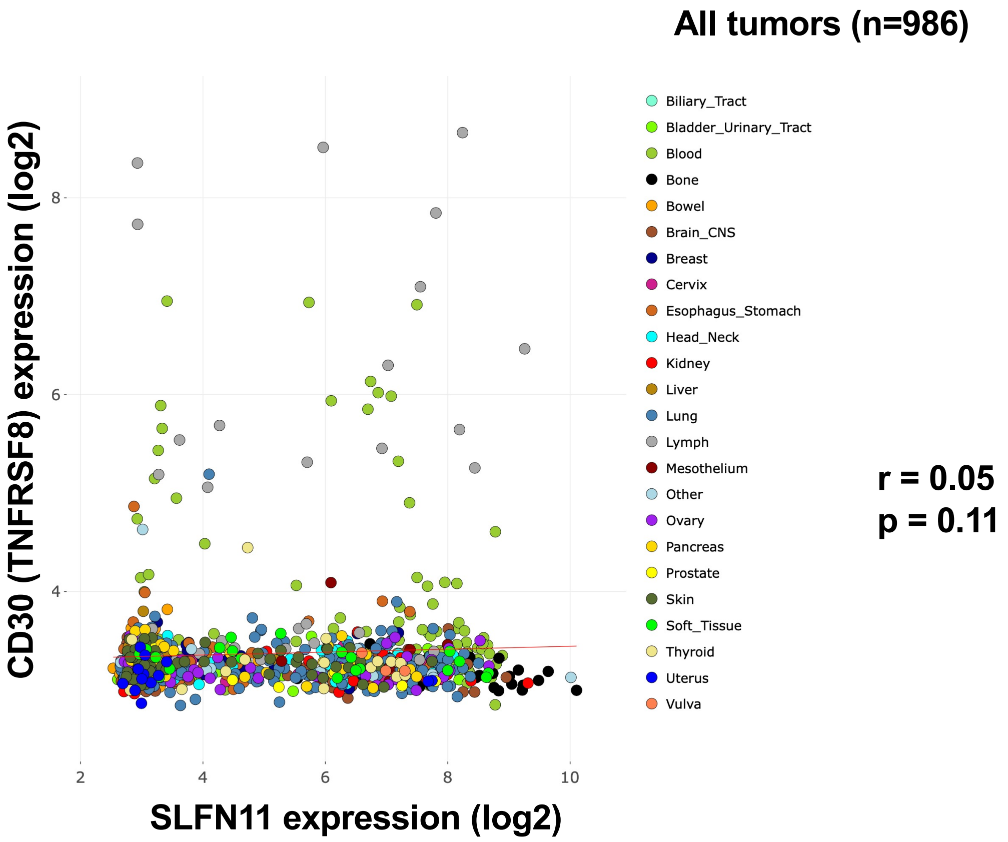

B

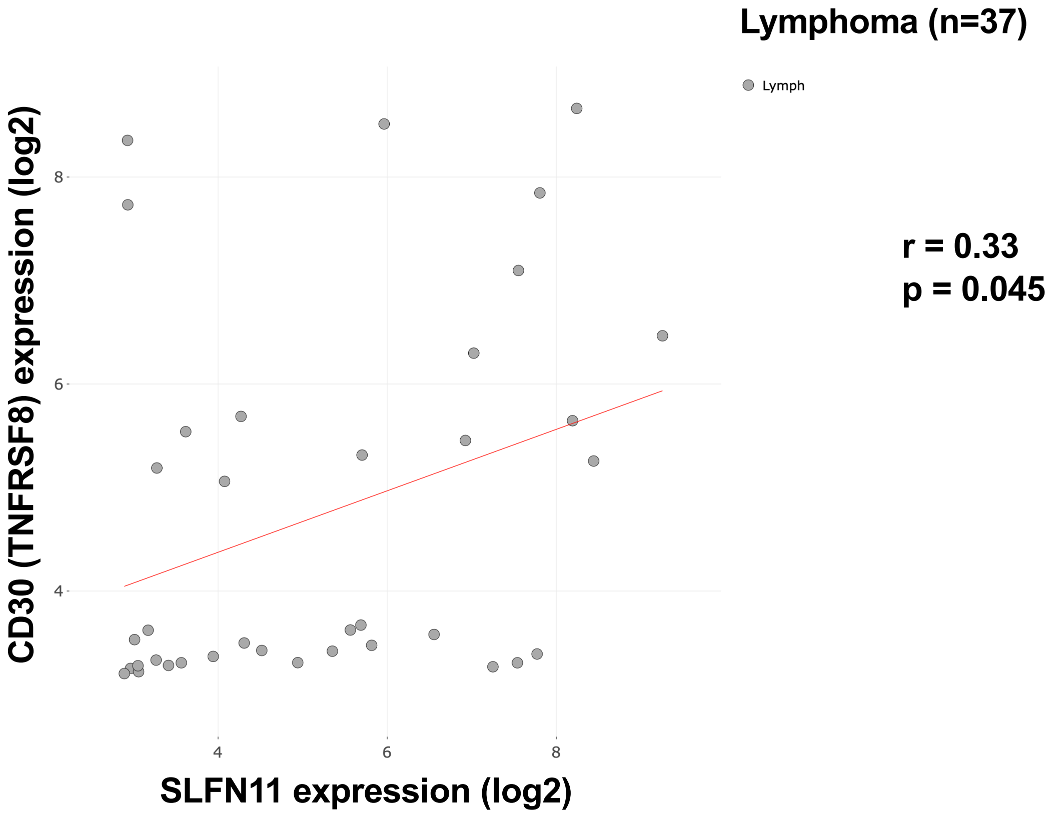

Supplemental Figure S4

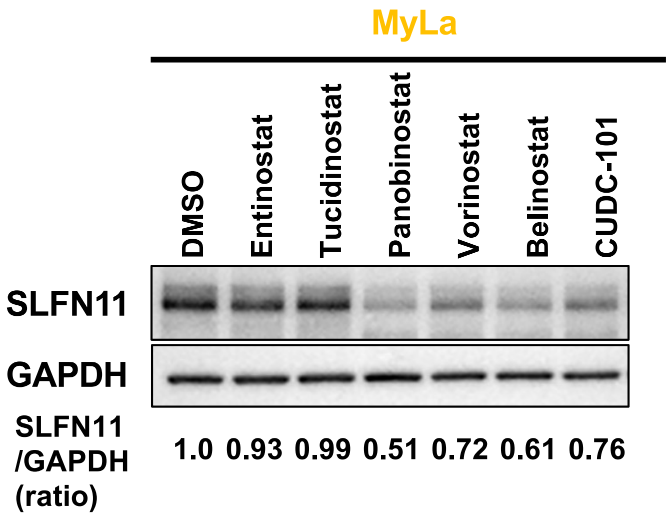

Supplement: zcaf037_Supplemental_Files [file zcaf037_supplemental_files.zip › Supplementary Figures_revised version.pdf]
