# Supplementary material for: Co-expression of CD30 and SLFN11 serves as a dual biomarker for the treatment of cutaneous T-cell lymphoma
Source: NAR Cancer. 2025 Oct 7;7(4):zcaf037. doi: 10.1093/narcan/zcaf037 (PMC12501776; doi:10.1093/narcan/zcaf037)
Supplement: zcaf037_Supplemental_Files [file zcaf037_supplemental_files.zip › Supplementary Table S1, figure S1-S4 legends.pdf]

Supplementary Table S1. Clinical characteristics obtained from enrolled MF/SS patients in this study

Supplementary Figure S1. SLFN11 expression in CD30$^+$ lymphoproliferative disorders and ATL. Representative images of immunohistochemical staining for CD30 and SLFN11 in skin-infiltrating tumor cells in patients with LyP, pcALCL, or ATL (original magnification, scale bar indicating 100 μm). By using an image analysis software (StrataQuest 7), % of SLFN11 positive (SLFN11$^+$) cells were quantified.

Supplementary Figure S2. Co-expression of SLFN11 and CD30 in CD30$^+$ lymphoproliferative disorders tumor cells. (**A**) Representative images of immunofluorescence staining for SLFN11, CD30, and DAPI (original magnification, scale bar indicating 100 μm). (**B**) An image cytometry analysis by StrataQuest 7 shows the intensity of SLFN11 and CD30 in each cell.

Supplementary Figure S3. Correlation between SLFN11 and CD30 expression in a public database across a variety of cell lines. (**A**) No significant correlation between SLFN11 and CD30 (TNFRSF8) mRNA expression among 986 cell lines in the Genomics of Drug Sensitivity in Cancer (GDSC) cell line database. (**B**) Significant correlation between SLFN11 and CD30 (TNFRSF8) mRNA expression among 37 lymphoma cell lines in the GDSC cell line database. The correlation coefficient (r) was calculated using the Pearson correlation coefficient test.

Supplementary Figure S4. Lack of SLFN11 induction by HDAC inhibitors in MyLa cells. Immunoblotting showing SLFN11 expression in MyLa cells treated with the indicated HDAC inhibitors (10 μM each) for 16 hours. GAPDH was used as a loading control. The relative ratio of SLFN11/GAPDH in each HDAC inhibitor-treated MyLa cell is indicated at the bottom. A representative result of two independent experiments is shown.
