# Supplementary material for: Co-expression of CD30 and SLFN11 serves as a dual biomarker for the treatment of cutaneous T-cell lymphoma
Source: NAR Cancer. 2025 Oct 7;7(4):zcaf037. doi: 10.1093/narcan/zcaf037 (PMC12501776; doi:10.1093/narcan/zcaf037)
Supplement: zcaf037_Supplemental_Files [file zcaf037_supplemental_files.zip › Supplemetary_Table_S1.revised version.pdf]

Supplementary Table S1. Clinical characteristics obtained from enrolled MF/SS patients in this study

Diagnosis	Case No.	Sex	Age	TNMB classification and stages	Type of skin manifestation	Serum LDH value	Serum sIL-2R value	% of SLFN11 positive cells	SLFN11 positivity	% of CD30 positive cells	CD30 positivity	Prebious systemic therapy performed before final staging	Systemic therapy performed after final staging	Survival time (months)	Outocome
MF	MF-3	F	61	Stage IA (T1bN0M0B0)	Plaque	188	207	9.645	-	1.91	-	none	none	105	Alive
MF	MF-1	M	75	Stage IA (T1bN0M0B0)	Plaque	171	367	19.185	+	38.81	+	none	none	117	Censored
MF	MF-2	F	36	Stage IA (T1bN0M0B0)	Plaque	N.D.	N.D.	13.555	+	40.85	+	none	IFN-γ	154	Alive
MF	MF-25	M	77	Stage IA (T1bN0M0B0)	Plaque	175	360	13.95	+	6.26	+	IFN-γ	Resminostat (Clinical trial)	73	Alive
MF	MF-5	M	62	Stage IB (T2bN0M0B0)	Plaque	185	280	4.32	-	18.4	+	none	Etretinate	111	Censored
MF	MF-6	F	66	Stage IB(T2bN0M0B0)	Plaque	190	268	6.805	-	3.58	-	none	IFN-γ	139	Alive
MF	MF-8	M	40	Stage IB(T2bN0M0B0)	Plaque (CD8)	146	260	5.29	-	4.78	-	none	none	123	Alive
MF	MF-7	M	68	Stage IB(T2bN0M0B0)	Plaque	207	482	21.145	+	2.25	-	Bexarotene	none	124	Died
MF	MF-24	M	48	Stage IB(T2bN0M0B0)	Plaque	185	227	35.81	+	24.57	+	none	none	172	Alive
MF	MF-21	M	62	Stage IIB (T3N0M0B0)	Tumor	175	473	3.465	-	0.74	-	none	none	132	Alive
MF	MF-10	F	67	Stage IIB (T3N0M0B0)	Tumor	185	401	5.845	-	63.47	+	none	IFN-γ, Etretinate, VP-16, Denileukin diftitox (Clinical trial), Bexarotene, Mogamulizumab	258	Died
MF	MF-22	M	51	Stage IIB (T3N0M0B0)	Tumor	153	465	7.19	-	6.86	+	none	IFN-γ, Bexarotene	96	Alive
MF	MF-16	M	80	Stage IIB (T3N0M0B0)	Tumor	222	326	1.965	-	92.57	+	none	IFN-γ, Bexarotene, Etretinate	77	Alive
MF	MF-14	M	60	Stage IIB (T3N0M0B0)	Tumor	204	966	22.52	+	1.43	-	none	Bexarotene	94	Alive
MF	MF-20	M	69	Stage IIB (T3N0M0B0)	Tumor	173	1617	40.52	+	0.18	-	none	Mogamulizumab	44	Alive
MF	MF-18	F	58	Stage IIB (T3N0M0B0)	Tumor CD30 LCT (CD8)	158	572	10	+	74.93	+	none	Bexarotene, Mogamulizumab, Vorinostat, Mogamulizumab+VP-16, Brentuximab vedotin	141	Alive
MF	MF-23	M	64	Stage IIIA (T4N0M0B0)	Plaque	200	820	50.915	+	7.16	+	none	IFN-γ	72	Alive
MF	MF-4	M	79	Stage IVA1 (T2bN0M0B2)	Plaque	242	1002	6.4	-	1.97	-	none	IFN-γ, Mogamulizumab	51	Alive
MF	MF-11	M	55	Stage IVA2 (T3N3M0B0)	Tumor	143	813	0.93	-	1.02	-	IFN-γ, Vorinostat, Denileukin diftitox, Mogamulizumab	Mogamulizumab, Bexarotene	30	Died
MF	MF-13	F	70	Stage IVA2 (T3N3M0B0)	Tumor	217	615	4.98	-	1.9	-	Bexarotene	Bexarotene	52	Censored
MF	MF-12	M	54	Stage IVA2 (T3N3M0B0)	Tumor	159	1443	53.61	+	10.81	+	none	THP-COP, VP-16, THP-COP, Steroid pulse, Mogamulizumab, VP-16, Bexarotene, THP-COP, Brentuximab vedotin	23	Died
MF	MF-17	M	68	Stage IVA2 (T3N3M0B0)	Tumor CD30 LCT	252	2032	30.69	+	25.27	+	Etretinate	Bexarotene, ABVD, Brentuximab vedotin	39	Died
MF	MF-9	F	73	Stage IVA2 (T2bN3M0B0)	Plaque CD30 LCT	262	693	16.15	+	53.35	+	IFN-γ	IFN-γ	11	Censored
MF	MF-19	F	57	Stage IVA2 (T3N3M0B0)	Tumor	215	887	12.625	+	25.27	+	THP-COP	Mogamulizumab, Bexarotene, VP-16, THP-COP, IFN-γ+Bexarotene, Mogamulizumab+VP-16, Mogamulizumab	128	Alive
MF	MF-15	M	65	Stage IVB (T3NxM1B0)	Tumor (CD8)	314	1299	6.32	-	0.2	-	IFN-γ, VP-16, Bexarotene, THP-COP, Mogamulizumab	THP-COP	2.54	Died
SS	SS-1	F	96	Stage IVA1 (T4NxM0B2)	Erythroderma	420	6135	5.865	-	20.21	+	none	none	2.19	Died
SS	SS-3	F	55	Stage IVA2 (T4N3M0B2)	Erythroderma	248	3422	30.88	+	6.07	+	none	Brentuximab vedotin, Bexarotene	17	Censored
SS	SS-5	M	78	Stage IVA1 (T4NxM0B2)	Erythroderma	432	1351	50.35	+	15.85	+	none	Mogamulizumab, Bexarotene	31	Died
SS	SS-2	M	65	Stage IVA1 (T4NxM0B2)	Erythroderma	208	756	15.89	+	14.24	+	IFN-γ, VP-16	Bexarotene, Mogamuloizumab	55	Died
SS	SS-4	M	58	Stage IVA2 (T4N3M0B2)	Erythroderma	213	1308	39.55	+	8.95	+	none	Mogamulizumab, VP-16, Bexarotene	18	Died

Abbreviations: MF, mycosis fungoides; SS, Sezary syndrome; F, female; M, male; LCT, large cell transformation; LDH, lactate dehydrogenase; sIL-2R, soluble interleukin-2 receptor
